# PCL/PHBV Microparticles as Innovative Carriers for Oral Controlled Release of Manidipine Dihydrochloride

**DOI:** 10.1155/2014/268107

**Published:** 2014-01-16

**Authors:** Fernanda Malaquias Barboza, Willian Moreira Machado, Luiz Renato Olchanheski Junior, Josiane Padilha de Paula, Sônia Faria Zawadzki, Daniel Fernandes, Paulo Vitor Farago

**Affiliations:** ^1^Laboratory of Pharmaceutical Products, Postgraduate Program in Pharmaceutical Science, Department of Pharmaceutical Sciences, State University of Ponta Grossa, 4748 Carlos Cavalcanti Avenue, 84030-900 Ponta Grossa, PR, Brazil; ^2^Laboratory of Cardiovascular Pharmacology, Postgraduate Program in Pharmaceutical Science, Department of Pharmaceutical Sciences, State University of Ponta Grossa, 4748 Carlos Cavalcanti Avenue, 84030-900 Ponta Grossa, PR, Brazil; ^3^Laboratory of Synthetic Polymers, Postgraduate Program in Chemistry, Department of Chemistry, Federal University of Paraná, Centro Politécnico Jardim das Américas, P.O. Box 19081, 81531-990 Curitiba, PR, Brazil

## Abstract

Microparticles of poly(**ε**-caprolactone) (PCL) and poly(3-hydroxybutyrate-*co*-3-hydroxyvalerate) (PHBV) containing manidipine dihydrochloride (MAN) were successfully prepared by the simple emulsion/solvent evaporation method. All formulations showed loading efficiency rates greater than 80% and average particle size less than 8 **μ**m. Formulations had spherical shape with smooth and porous surface for PCL and PHBV, respectively. According to Fourier-transform infrared spectroscopy, initial components were not chemically modified during microencapsulation. X-ray diffraction patterns and differential scanning calorimetry demonstrated that this process led to drug amorphization. *In vitro* dissolution studies showed that all microparticles prolonged MAN release, mainly which one obtained using PCL that contained 5% of drug loaded (*PCL-M5*). Animal studies demonstrated that formulation *PCL-M5* was able to keep the variation of mean arterial pressure after phenylephrine administration up to 24 hours. These data confirmed the sustained antihypertensive effect of the investigated microparticles. Results provided an experimental basis for using formulation *PCL-M5* as a feasible carrier for oral controlled release of MAN intended for treating high blood pressure.

## 1. Introduction

The primary goal of the treatment of hypertensive patients is to achieve the maximum decrease in the long-term total risk of cardiovascular and renal morbidity and mortality. In addition to the lowering of blood pressure, a successful drug also requires appropriate pharmacological properties in order to avoid comorbidities [1, 2].

**Figure 1:**
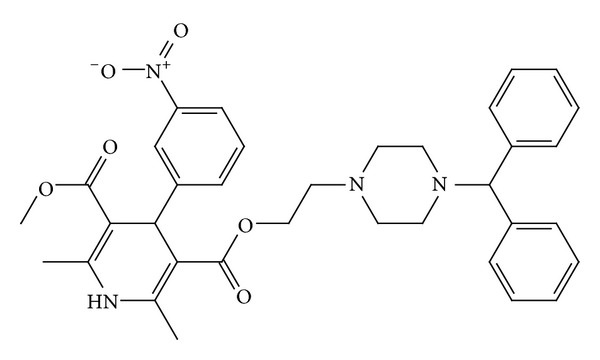
Chemical structure of manidipine.

Among several antihypertensive drugs, calcium channel blocks are widely used due to their efficacy, tolerability, and safety. In this group, manidipine, 2-[4-(diphenylmethyl)-1-piperazinyl]ethylmethyl(±)-1, 4-dihydro-2, 6-dimethyl-4-(m-nitrophenyl)-3, 5-pyridinedicarboxylate (Figure 1), stands out as a third-generation calcium channel blocker, effective for treating high blood pressure even to elderly people or patients with associated diseases such as diabetes and nephropathy [3–5].

In particular, it presents high tolerability which results in a low frequency of adverse events. Several studies have reported that manidipine has a low oedematogenic potential when compared to other dihydropyridines and it is not associated with increased heart rate. Furthermore, this drug does not appear to activate the sympathetic nervous system [6]. In addition to reducing arterial blood pressure, manidipine has been related to metabolic effects of potential clinical interest. It blocks T-type calcium channels present in the efferent glomerular arterioles that can decrease intraglomerular pressure and microalbuminuria [2].

As other dihydropyridine derivatives, manidipine exhibits high clearance and first pass metabolism and hence a low systemic bioavailability. Moreover, after oral administration, it presents a peak plasma concentration after 1-2 h with an apparent elimination half-life of 4–8 h [7].

In spite of the fact that manidipine presents a short elimination half-life, its high lipophilicity makes it being rapidly removed from circulation to adipose tissue. Manidipine is then continuously released from fat cells which promotes a time of action up to 24 hours [4]. This spontaneous compartmentalization becomes a complicating factor for drug therapy, since drug release is dependent on the mass of adipose tissue of the patient, which is very changeable in population. Therefore, manidipine demonstrates a sustained but heterogeneous and individualized release profile.

Thus, a further improvement is necessary to attain a substantial advance in absorption and bioavailability of manidipine [7, 8]. In that sense, the development of new formulations useful for controlling manidipine release along gastrointestinal tract and enhancing drug therapy [9] is required.

Considering this purpose, microencapsulation is able to avoid its fast compartmentalization in adipose cells, besides showing high efficiency over controlling drug delivery and protecting drug degradation by physiological metabolism and adverse environmental conditions [10–15].

Few studies describe delivery systems for manidipine. Papers are only devoted to describe manidipine complexation using cyclodextrin and its derivatives [7, 8]. The literature does not report works involving manidipine-loaded polymeric microparticles. Thus, the aim of this study was to obtain poly(**ε**-caprolactone) (PCL) and poly(3-hydroxybutyrate-*co*-3-hydroxyvalerate) (PHBV) microparticles containing manidipine dihydrochloride. These polyesters are attractive materials for controlled-release drug applications due to their biocompatibility and biodegradability since their decomposition products are not toxic. Manidipine dihydrochloride-loaded PCL/PHBV microparticles were also characterized and *in vitro* and *in vivo* studies were carried out in order to explore their potential as oral drug delivery carriers intended for treating high blood pressure.

## 2. Materials and Methods

### 2.1. Materials

Manidipine dihydrochloride (MAN) (Zi Bo Riyuexin Chemical Industrial, Shandong, China), poly(**ε**-caprolactone) (PCL) (*M*_*w*_ = 70,000–90,000 g·mol^−1^, Sigma-Aldrich, St. Louis, MO, USA), poly(3-hidroxybutirate-*co*-3-hydroxyvalerate) (PHBV) (*M*_*w*_ = 380,000 g·mol^−1^, 8.70 mol% hydroxyvalerate, Biocycle L110, PHB Industrial, Serrana, Brazil), polysorbate 80 (Sigma-Aldrich, St. Louis, MO, USA), and poly(vinyl alcohol) (PVA) (*M*_*w*_ = 72,000 g·mol^−1^, 88.5 mol% of hydrolysis, Vetec, Rio de Janeiro, Brazil) were used as received. All reagents and solvents were of analytical grade.

### 2.2. Preparation of PCL and PHBV Microparticles

Polymeric microparticles containing MAN were prepared by simple emulsion/solvent evaporation method. Dichloromethane and chloroform were used as organic solvent for PCL and PHBV, respectively. The emulsion was stabilized using PVA and polysorbate 80. Two different formulations (Table 1) were obtained for each polymer (PCL and PHBV) depending on the theoretical amount of MAN used in their composition (5 and 10%). Unloaded microparticles were also prepared as negative controls (*PCL-M0 e PHBV-M0*). All formulations were obtained in triplicate.

In brief, the organic phase was quickly added into the aqueous phase under mechanical stirring (3500 rpm) for 5 min. The emulsion was kept under mechanical stirring (1000 rpm) at room temperature for 4 h. After solvent evaporation, microparticles were separated by centrifugation (3500 rpm), washed twice with purified water, and dried at 35 ± 5°C for 48 h. All procedures were performed under dark conditions.

In order to provide a comparative analysis, physical mixtures between MAN and the chosen polyesters were also prepared using 1 : 1 (MAN : polymer) in weight proportion.

### 2.3. Determination of Loading Efficiency

The amount of drug loaded into PCL and PHBV microparticles was determined by means of a previously developed method by high performance liquid chromatography with UV detection (HPLC/UV). An amount of microparticles, equivalent to 50 mg of MAN, was weighted and magnetic stirred with 100 mL of methanol for 12 h in order to completely extract the drug from microparticles. Samples were suitable diluted in methanol and filtered through a poly(vinylidene fluoride) membrane filter (Durapore membrane, 0.45 **μ**m pore size, Millipore, Bedford, MA, USA). The concentration of MAN was obtained chromatographically through a LiChroCart (Merck, Darmstadt, Germany) analytical column (4 × 250 mm) filled with LiChrospher 100 RP-18 (5 *μ*m) with UV detection at 265 nm in triplicate. The mobile phase was composed of phosphate buffer pH 5.0 and acetonitrile (9 : 1 v/v) with a flow rate of 0.5 mL·min^−1^.

The amount of manidipine was calculated and reported as loading efficiency, following (1)

(1) Consider  loading  efficiency% =mass  of  MAN  in  microparticlestheoretical  mass  of  MAN×100.

### 2.4. Characterization

#### 2.4.1. Analyses of Morphology and Surface

Morphology and surface data were evaluated using a scanning electron microscope (SSX-550 Superscan, Shimadzu, Kyoto, Japan). Samples were mounted on aluminum stubs, sputtered with gold (IC-50 Ion Coater, Shimadzu, Kyoto, Japan). Micrographs were obtained at an accelerating voltage of 10 kV with different magnifications.

Microparticles surface was analyzed by wide-angle X-ray powder diffraction in an X-ray diffractometer (Shimadzu XRD-6000, Kyoto, Japan) in order to observe peaks related to crystalline structures [16]. Samples were scanned from a Cu-K*α* source (*λ* = 1.5418 Å) at 40 kV and 30 mA, using 2**θ** from 2° to 80° at a scan rate of 2°·min^−1^.

#### 2.4.2. Determination of Particle Size and Granulometric Distribution

The particle size distribution was estimated from the measurement of about 200 particles, assuming spherical shape, observed in an arbitrary chosen area in enlarged micrographs by Image Tool Software (3.0 version, San Antonio, TX, USA). Span, a mathematical value related to the granulometric dispersion, was calculated using (2):

(2) $$\begin{array}{c} \mathrm{span}=\frac{d_{\left(v,90\right)}-d_{\left(v,10\right)}}{d_{\left(v,50\right)}}, \end{array}$$

where *d*_(*v*,10)_, *d*_(*v*,50)_, and *d*_(*v*,90)_ match, respectively, to the particles diameter at 10, 50, and 90% of the accumulated distribution of the sample.

#### 2.4.3. Fourier-Transform Infrared Spectroscopy

Raw materials, PCL, and PHBV microparticles and physical mixtures were analyzed by Fourier-transform infrared (FTIR) spectroscopy on a Shimadzu IR Prestige-21 spectrophotometer (Kyoto, Japan). Samples were recorded from 4000 to 400 cm^−1^, using KBr pellets filled with 1% of each sample using 64 scans·min^−1^ and resolution of 2 cm^−1^.

#### 2.4.4. Thermal Analyses

The thermal stability of PCL and PHBV microparticles was investigated with a TGA209 thermobalance (Netzsch-Gerätebau, Selb, Germany) using 5 mg of sample into platinum crucibles. The thermogravimetric analyses (TGA) were held under dynamic N_2_ atmosphere with a flow rate of 50 mL·min^−1^. Temperature ranged from 25 to 600°C, following a constant heating rate of 10°C·min^−1^.

Differential scanning calorimetry (DSC) curves were obtained in a DSC-60 calorimeter (Shimadzu, Kyoto, Japan) using aluminum crucibles with 5 mg of sample, under dynamic N_2_ atmosphere with a flow rate of 50 mL·min^−1^. Temperature ranged from −120 a 250°C, with a constant rate of 10°C·min^−1^, according to the particular characteristics of each material. The equipment was previously calibrated with indium (m.p. = 156.6°C; Δ*H*_melting_ = 28.54 J·g^−1^) and zinc (m.p. = 419.6°C). Thermograms provided information about thermal behavior changes of the studied materials [16, 17].

### 2.5. *In Vitro* Release Studies

Dissolution rates of MAN as pure drug and from polyester microparticles were carried out in a Nova Ética 299-6 ATTS dissolution tester equipped with paddles. Systems were kept at a thermostatically controlled temperature of 37 ± 0.5°C and stirred at 50 rpm. The chosen dissolution medium was acetate buffer (50 mM, pH 4.0, 900 mL) as recommended by The Japanese Pharmacopoeia [18]. All experiments were performed under dark conditions.

At fixed time intervals, samples were collected, filtered (0.45 *µ*m pore size) and analyzed spectrophotometrically at 228 nm [18]. The dissolution value was obtained from the amount of drug released. A correction factor was applied to the cumulative dilution caused by replacement of the sample with an equal volume of fresh medium.

#### 2.5.1. Analysis of Release Behavior

In order to compare the dissolution profiles of MAN and its microparticulate systems, model-independent and model-dependent methods were performed as summarized in Table 2.

As model-independent analysis, dissolution efficiency, the area under a dissolution curve between defined time points [19], was used to compare dissolution profiles. One-way ANOVA with Tukey's *post hoc* test was also used to compare release rates of the pure drug and polymeric microparticles [20] by Microsoft Excel 2007 software (Salt Lake City, UT, USA). A *P* value of ≤0.05 was used to indicate statistically significant differences.

Profiles were also investigated by model-dependent approaches [21, 22] using the MicroMath Scientist 2.01 software (Salt Lake City, USA). Data were tested to fit first-order, biexponential, zero-order, weibull, and monolag equations (Table 2). For selecting the best model of MAN releasing, it was considered the correlation coefficient (*r*), the model selection criteria (MSC), and graphical adjustment.

### 2.6. *In Vivo* Animal Studies of Antihypertensive Potential

Female Wistar rats, 90 days old, weighting between 200 and 300 g, were housed at room temperature (22 ± 2°C) with controlled light/dark cycle, with free access to water and food. All procedures were performed in agreement with the Ethics Committee of State University of Ponta Grossa in compliance to the Guide for the Care and Use of Laboratory Animals published by the US National Institutes of Health.

#### 2.6.1. Experimental Protocol

Ninety-six rats were randomly divided into four groups of twenty-four animals each one. Group 1 received pure MAN (1 mg·kg^−1^, orally), group 2 received *PCL-M5* microparticles (amount equivalent to 1 mg of the drug·kg^−1^, orally), group 3 received *PHBV-M5 *microparticles (amount equivalent to 1 mg of the drug·kg^−1^, orally), and group 4 received sterile water (500 *μ*L). At different time intervals after treatment (1, 2, 6, 12, and 24 h) animals were instrumented for blood pressure measurement, as described below. Mean blood pressure and vasoconstrictor response to phenylephrine were recorded.

#### 2.6.2. Evaluation of Cardiovascular Parameters

Under anesthesia (ketamine, 75 mg·kg^−1^, and xylazine 15 mg·kg^−1^), polyethylene cannula (PE-50) was inserted into the left femoral vein of the rats for phenylephrine injections (10 nmol·kg^−1^). Then, the right carotid artery was isolated and a heparinized polyethylene catheter (Angiocath, number 19, BD, Curitiba, Brazil) was inserted for measuring blood pressure. Mean arterial blood pressure (mmHg) were recorded by integration software (Chart 7 pro, ADInstruments, Dunedin, New Zealand). Finally, rats were sacrificed using an anesthetic overdose. Statistical analysis was performed using one-way ANOVA followed by Dunnet's *post hoc* test. A *P* value of ≤0.05 was used to indicate statistically significant differences.

## 3. Results and Discussion

### 3.1. Preparation of PCL and PHBV Microparticles

Polyester microparticles were successfully obtained by the proposed simple emulsion/solvent evaporation procedure, resulting in suitable yield rates (over 80%) for all formulations.

### 3.2. Determination of Loading Efficiency

Loading efficiencies obtained for PCL and PHBV microparticles using HPLC/UV method are summarized in Table 3. All formulations presented high loading rates, greater than 80%. These results can be related to the low water solubility of MAN (<1 mg·mL^−1^ at 25°C) which prevents its migration to the external aqueous phase and provides high drug entrapment into the microparticles.

### 3.3. Characterization

#### 3.3.1. Analyses of Morphology and Surface

**Figure 2:**
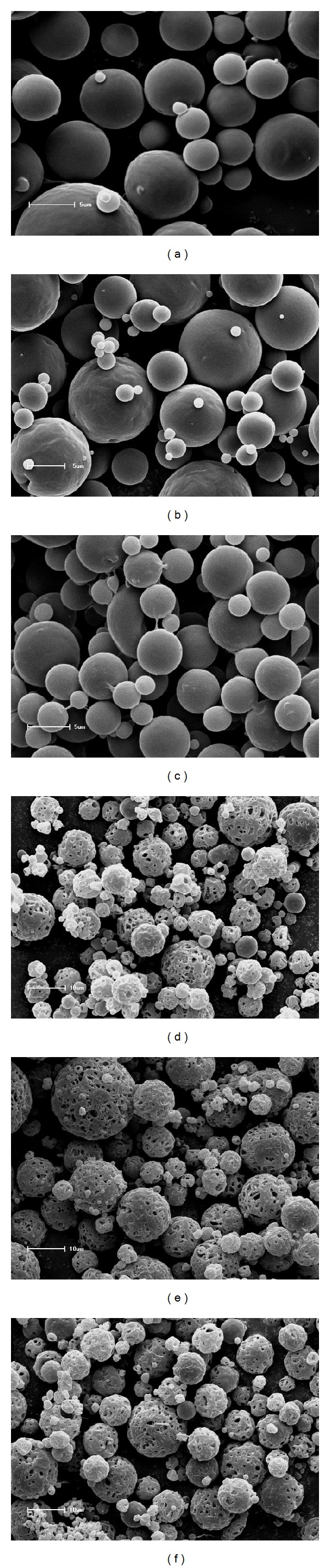
SEM photomicrographs of PCL and PHBV microparticles: *PCL-M0* (a), *PCL-M5 *(b), *PCL-M10* (c), *PHBV-M0 *(d), *PHBV-M5 *(e), and* PHBV-M10* (f).

SEM micrographs showed that PCL microparticles presented a spherical shape with a smooth and regular surface (Figures 2(a)–2(c)). PHBV microparticles also showed a spherical shape, however, with a porous surface (Figures 2(d)–2(f)). Pores were observed even on the negative control. The presence of pores represents morphological evidence that can change the drug release process from microparticles. According to the literature, pores may result from the nature of the polyhydroxyalkanoate used or from fast removal of the organic solvent [23].

**Figure 3:**
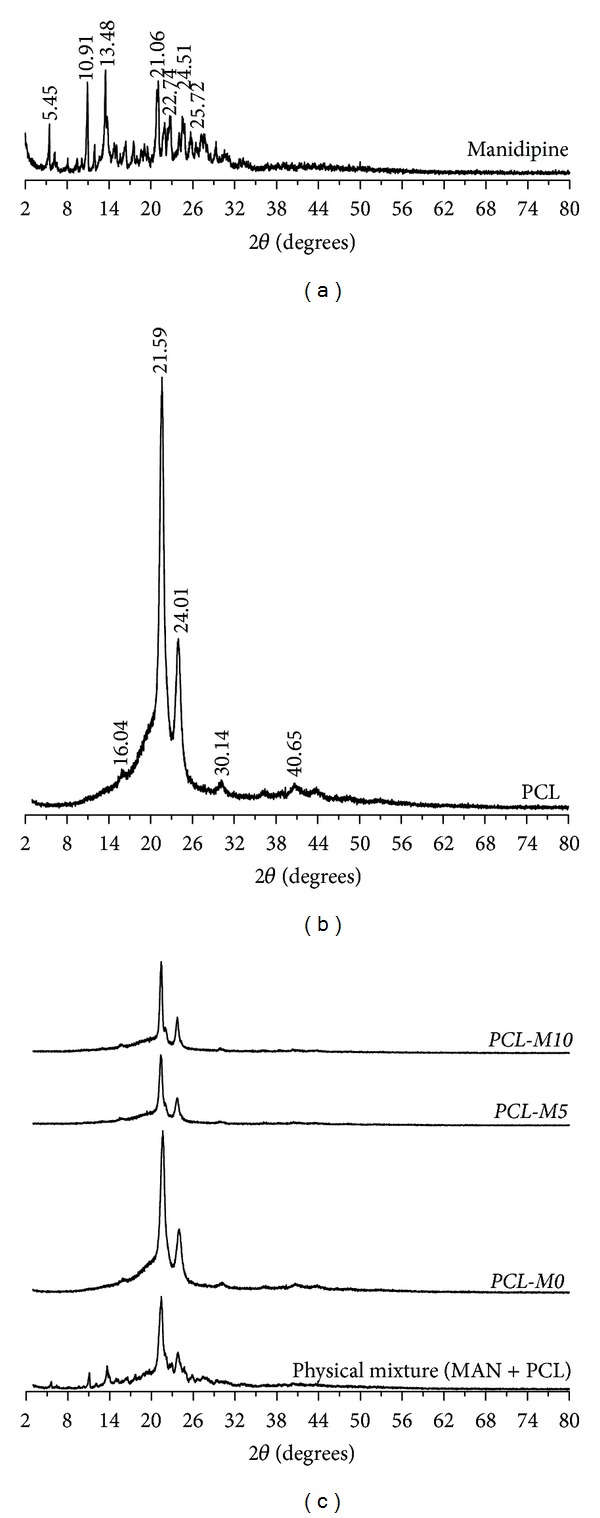
X-ray diffractions spectra of MAN, PCL, physical mixture (MAN + PCL), and PCL microparticles.

**Figure 4:**
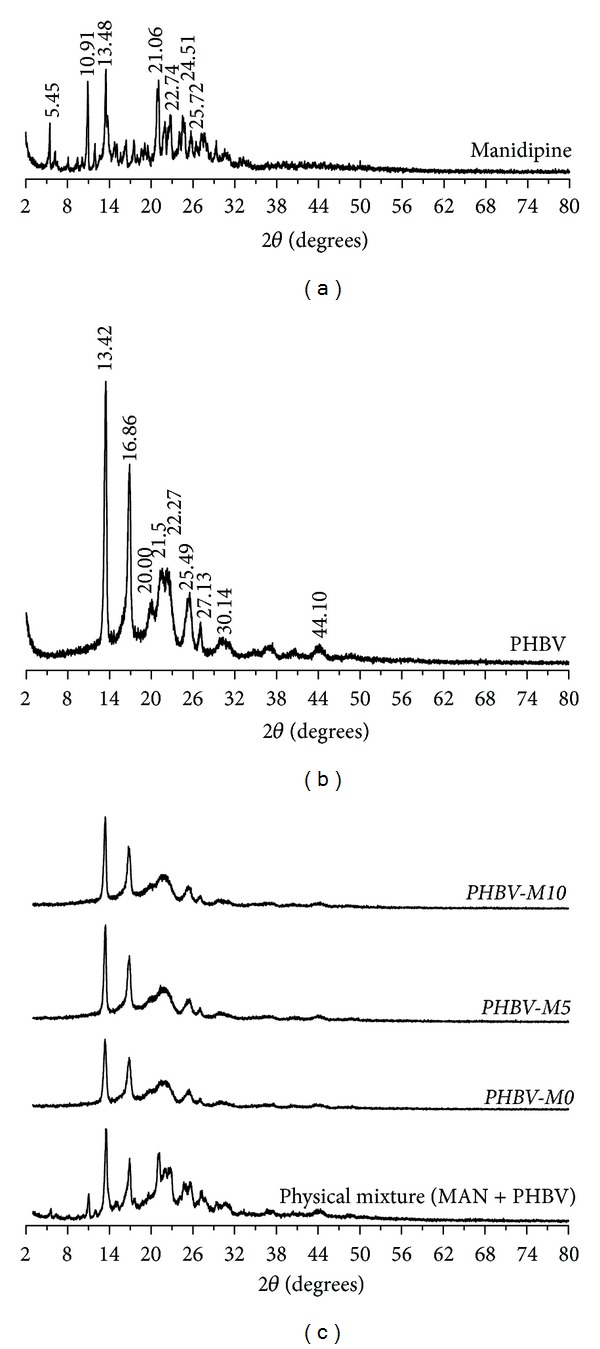
X-ray diffractions spectra of MAN, PHBV, physical mixture (MAN + PHBV), and PHBV microparticles.

X-ray diffraction patterns are indicated in Figures 3 and 4. Different peaks related to a crystalline structure were observed either to MAN or polymers. PCL and PHBV formulations showed crystalline diffraction patterns very similar to pure polyesters. These results suggest that the microencapsulation procedure provided a remarkable decrease of the crystalline diffraction peaks of manidipine leading to drug amorphization. In general, amorphous solids dissolve more quickly than crystalline forms, due to free energies involved in their dissolution process. Solids in amorphous state have randomly arranged molecules; thus, low energy is required to separate them what makes their dissolution faster when compared to crystalline form [24].

#### 3.3.2. Determination of Particle Size and Granulometric Distribution

The particle size and granulometric dispersion (span) obtained for PCL and PHBV microparticles are indicated in Table 4. All formulations presented mean diameters values under 8 *μ*m. These micrometer-sized particles can provide a prolonged effect of MAN, since they present an extended intestinal transit [25].

Regarding span, most of the microparticles showed values under 2 which represent that they have a narrow dispersion around the mean particle size, suggesting a suitable unimodal behavior.

#### 3.3.3. Fourier-Transform Infrared Spectroscopy

**Figure 5:**
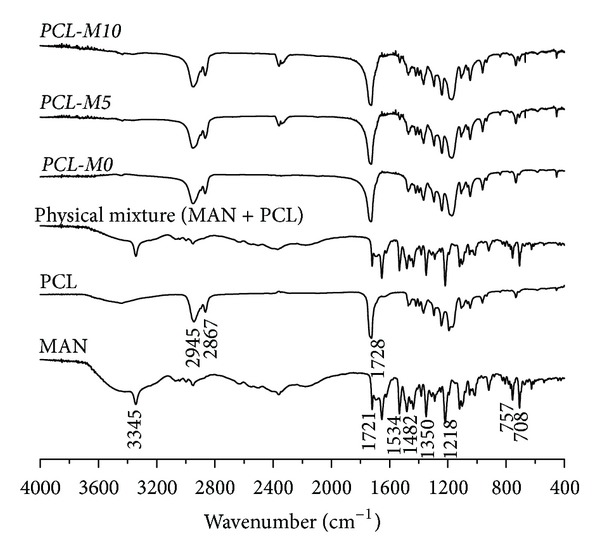
FTIR spectra of MAN, PCL, physical mixture (MAN + PCL), and PCL microparticles.

**Figure 6:**
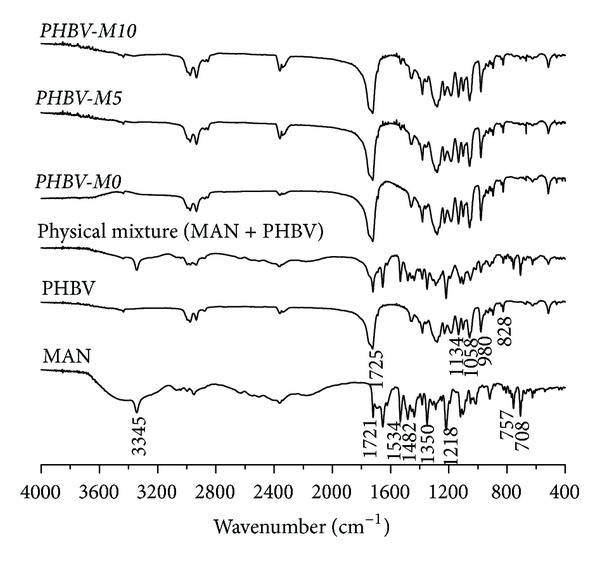
FTIR spectra of MAN, PHBV, physical mixture (MAN + PHBV), and PHBV microparticles.

FTIR spectra obtained for MAN, PCL, PHBV, physical mixtures, and PCL and PHBV microparticles are shown in Figures 5 and 6.

The FTIR spectrum of pure MAN presented a typical N–H stretching band at  3345 cm^−1^, ester C=O stretching band at 1721 cm^−1^, NO_2_ asymmetric and symmetric stretching bands at 1534 and 1350 cm^−1^, aromatic C=C stretching band at 1482 cm^−1^, C–N stretching band at 1218 cm^−1^, and out-of-plane bending of aromatic C–H bonds at 757 and 708 cm^−1^.

As PCL is an aliphatic polyester, its spectrum showed a strong band at 1728 cm^−1^, corresponding to ester C=O stretching and two bands at 2945 and 2867 cm^−1^ assigned to symmetric and asymmetric C–H_2_ stretching, respectively [26]. Similarly, PHBV spectrum exhibited a strong band at 1725 cm^−1^ due to ester C=O stretching. Its symmetric and asymmetric –C–O–C– stretching vibration showed typical bands from 828 to 980 cm^−1^ and 1058 to 1134 cm^−1^, respectively [23].

Physical mixtures and MAN-loaded microparticles presented band assignments at the same wavenumber range of FTIR spectra and formulations did not show any new band assignment. Both polymers used have terminal primary hydroxyl groups, whereas MAN presents a reactive aromatic nitro group. Aromatic nitro compounds are usually reduced to anilines. However, during microencapsulation, there is no reducing medium to make it possible.

Therefore, it is suitable to ensure that initial components were not chemically modified, safely preserving the expected therapeutic effect of the drug.

#### 3.3.4. Thermal Analyses

Thermogravimetric analyses (TGA) of MAN showed two events of mass loss, the first event between 166 and 203°C (Δ*m* = 2.99%) and the second one between 208 and 522°C (Δ*m* = 87.46%). On the other hand, TGA curves of polymers presented a single event of mass loss at the temperature range of 247 and 467°C (Δ*m* = 97.84%) and 224 and 291°C (Δ*m* = 96.91%) for PCL and PHBV, respectively.

PCL microparticles started its mass loss at 224°C while PHBV microparticles started it at 161°C. These results indicate that PCL formulations are more thermally stable than PHBV formulations. Similar results have been described in the literature for PCL and PHBV microparticles [24, 27].

DSC curves demonstrated that MAN showed a sharp endothermic event at 210°C. PCL and PHBV presented their melting temperatures at 60 and 168°C, respectively. All PCL and PHBV microparticles had a single melting event at the same temperature observed for pure polymers and the typical melting event of MAN was not verified in DSC curves of PCL and PHBV formulations. Therefore, these results confirmed the previously obtained data from X-ray diffraction which suggested the drug amorphization.

### 3.4. *In Vitro* Release Studies

**Figure 7:**
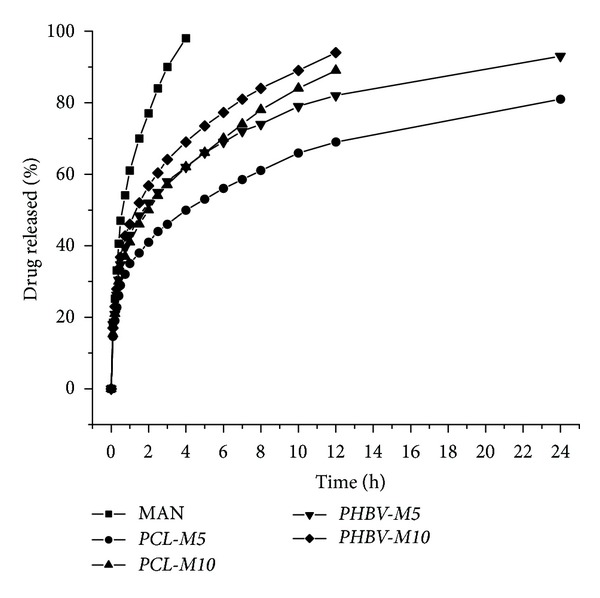
Release profiles of MAN and MAN-loaded microparticles into acetate buffer (50 mM, pH 4.0).

Dissolution rates of MAN and MAN-loaded microparticles are shown in Figure 7. Release profiles demonstrated that the mean time for 80% releasing of pure drug was about 2.2 hours. However, PCL microparticles presented mean dissolution times of 23.0 hours (*PCL-M5*) and 8.7 hours (*PCL-M10*) for 80% drug release. For PHBV formulations, a value of 80% drug release was achieved in mean dissolution times of 10.7 and 6.7 hours for *PHBV-M5* and *PHBV-M10*, respectively. These results confirm that all formulations exhibited a prolonged release of the drug.

Formulations containing 5% of MAN prolonged the drug release for a longer time when compared to the other ones with 10%. This result can be justified because of the increased polymer/drug proportion into *M5* formulations. In addition, the presence of pores in the surface of PHBV microparticles, as previously verified in the SEM micrographs, can make the solvent access easier and is strongly related to faster dissolution profiles. Regarding the obtained drug release profiles, *PCL-M5* and *PHBV-M5* were the best formulation for providing a controlled drug release and was chosen for further *in vivo* pharmacological evaluation.

#### 3.4.1. Analysis of Release Behavior

Regarding dissolution efficiency (DE) data, PCL and PHBV microparticles reduced the dissolution rate, as expected for all controlled release formulations. Whereas the pure drug presented a dissolution efficiency of 73.6%, along four hours, PCL and PHBV formulations showed 39.7 and 48.1% for *PCL-M5* and *PCL-M10,* and 49.5 and 54.0% for *PHBV-M5* and *PHBV-M10*, respectively.

For the aim of evaluating the difference among release profiles, the Tukey's *post hoc* test was performed on the results of ANOVA. This analysis indicated a statistically significant difference between pure drug and *PCL-M5 *(*P* = 0.001), *PCL-M10 *(*P* = 0.025), and *PHBV-M5 *(*P* = 0.023). However, there was no statistical difference between MAN and *PHBV-M10* (*P* = 0.099).

Release profiles were also fitted to mathematical models and the selection of the best model considered *r*, MSC, and the graphic adjustment. Both MAN and PCL and PHBV microparticles were better fitted to the biexponential equation (Table 5) than other models. The burst release apparent rate constants (*α*) and the slow release apparent rate constants (*β*) are reported in Table 5.

All results demonstrated that PCL and PHBV microparticles were able for controlling MAN release, nevertheless without changing its release model. In that sense, it is possible to predict that formulations just modified undesirable pharmacokinetic characteristics of the drug with no interference in its releasing mechanism throughout the gastrointestinal tract.

### 3.5. *In Vivo* Animal Studies of Antihypertensive Potential

**Figure 8:**
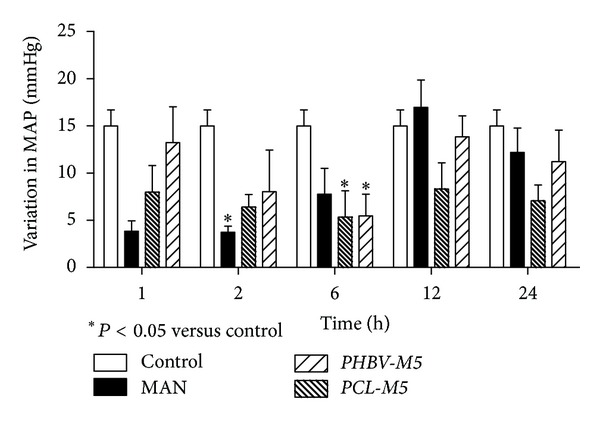
Effects of MAN on mean arterial pressure (MAP) after phenylephrine injection. Formulations *PCL-M5* and *PHBV-M5*, pure MAN, and water (control group) were administered orally to the animals. Statistical analysis was performed using ANOVA test followed by Dunnet's *post hoc* test.

Considering the previously reported results, both *PCL-M5* and *PHBV-M5* were chosen for further pharmacological investigation. *In vivo* performances of MAN-loaded microparticles over mean arterial pressure (MAP) in rats are shown in Figure 8.

For all studied times, control group showed a variation in MAP of 14.99 mmHg forward phenylephrine administration. One, 2, 6, 12, and 24 hours after phenylephrine administration, *PCL-M5* formulation demonstrated changes in MAP of 7.98, 6.40, 5.33, 8.33, and 7.07 mmHg, respectively. *PHBV-M5* presented a variation in MAP of 13.24, 8.04, 5.46, 13.84, and 11.22 mmHg, respectively, whereas pure MAN had changes in MAP of 3.85, 3.74, 7.77, 16.97, and 12.20 mmHg, respectively. A low variation in MAP forward phenylephrine administration demonstrates that the antihypertensive drug is able to keep the basal arterial pressure under any stimulus.

Therefore, formulation *PCL-M5* was able to hold down the mean arterial pressure variation up to 24 hours in contrast to that observed for the pure drug and *PHBV-M5*. These *in vivo* data provided an experimental basis for using formulation *PCL-M5* as a feasible carrier for oral controlled release of MAN intended for treating high blood pressure.

## 4. Conclusions

The preparation of PCL and PHBV microparticles containing MAN was reported for the first time. The formulations combined suitable physicochemical characteristics and prolonged drug release. Morphological and surface data played a crucial role to explain drug release performance. Animal studies demonstrated that formulation *PCL-M5* was able to hold down the variation in mean arterial pressure up to 24 hours and thereby provided a long-lasting antihypertensive effect. In summary, this formulation is a feasible carrier for controlled release of MAN and can be used into further innovative medicines intended for treating high blood pressure.

## Figures and Tables

**Table 1:** Composition of manidipine-loaded and unloaded PCL/PHBV microparticles.

Formulation	*PCL-M0*	*PCL-M5*	*PCL-M10*	*PHBV-M0*	*PHBV-M5*	*PHBV-M10*
Organic						
MAN (g)	—	0.1	0.2	—	0.1	0.2
PCL (g)	2.0	1.9	1.8	—	—	—
PHBV (g)	—	—	—	2.0	1.9	1.8
Dichloromethane (mL)	40	40	40	—	—	—
Chloroform (mL)	—	—	—	40	40	40
Aqueous						
2% PVA solution (mL)	200	200	200	200	200	200
Polysorbate 80 (g)	0.25	0.25	0.25	0.25	0.25	0.25
NaOH 0.1 M (mL)	2	2	2	—	—	—

**Table 2:** Mathematical models applied to dissolution experiments.

Model	Equation
Dissolution efficiency	$\text{DE}=\frac{\int_{0}^{t}y\cdot dt}{y_{100}\cdot t}\times 100\%$
First-order	%*D* = 100(1 − *e* ^−*kt*^)
Biexponential	%*D* = 100[1 − (*Ae* ^−*αt*^ + *Be* ^−*βt*^)]
Zero-order	%*D* = *kt*
Weibull	%*D* = 100[1 − *e* ^−(t/TD)*b*^]
Monolag	%*D* = 100[1 − *e* ^−*k*(*t*−*x*)^]

%*D*: dissolved percentage; *b*: shape parameter; TD: time interval necessary to release 63.2% of the drug; *k*, *α*, and *β*: kinetics constants; *t*: dissolution time; *A* and *B*: initial drug concentrations that contribute for the two dissolution stages.

**Table 3:** Loading efficiency values obtained to MAN-loaded PCL and PHBV microparticles.

Formulation	Loading efficiency (%) ± SD*
*PCL-M5*	88.99 ± 1.46
*PCL-M10*	89.79 ± 2.88
*PHBV-M5*	85.29 ± 2.28
*PHBV-M10*	90.98 ± 2.43

*SD: standard deviation.

**Table 4:** Particle size and granulometric size distribution of PCL and PHBV formulations.

Formulation	Average particle size (*μ*m)	Span
*PCL-M0*	5.69	1.52
*PCL-M5*	6.49	1.73
*PCL-M10*	6.74	2.21
*PHBV-M0*	6.04	1.17
*PHBV-M5*	7.66	1.79
*PHBV-M10*	4.10	1.40

**Table 5:** Release data from MAN and MAN-loaded microparticles.

Biexponential model				
	MSC	*R*	*α* (h^−1^)	*β* (h^−1^)
MAN	2.7983	0.9930	0.8703	0.7732
PCL-M5	4.7431	0.9973	2.5659	0.0619
PCL-M10	4.7644	0.9979	1.5495	0.1234
PHBV-M5	4.8927	0.9988	1.8285	0.0959
PHBV-M10	5.8751	0.9992	2.3060	0.1698
